# Functional biomaterials for tendon/ligament repair and regeneration

**DOI:** 10.1093/rb/rbac062

**Published:** 2022-09-05

**Authors:** Yunkai Tang, Zhen Wang, Lei Xiang, Zhenyu Zhao, Wenguo Cui

**Affiliations:** Department of Orthopaedics, Shanghai Key Laboratory for Prevention and Treatment of Bone and Joint Diseases, Shanghai Institute of Traumatology and Orthopaedics, Ruijin Hospital, Shanghai Jiao Tong University School of Medicine, Shanghai 200025, P. R. China; Department of Orthopaedics, Shanghai Key Laboratory for Prevention and Treatment of Bone and Joint Diseases, Shanghai Institute of Traumatology and Orthopaedics, Ruijin Hospital, Shanghai Jiao Tong University School of Medicine, Shanghai 200025, P. R. China; Department of Orthopaedics, Shanghai Key Laboratory for Prevention and Treatment of Bone and Joint Diseases, Shanghai Institute of Traumatology and Orthopaedics, Ruijin Hospital, Shanghai Jiao Tong University School of Medicine, Shanghai 200025, P. R. China; Department of Orthopaedics, Shanghai Key Laboratory for Prevention and Treatment of Bone and Joint Diseases, Shanghai Institute of Traumatology and Orthopaedics, Ruijin Hospital, Shanghai Jiao Tong University School of Medicine, Shanghai 200025, P. R. China; Department of Orthopaedics, Shanghai Key Laboratory for Prevention and Treatment of Bone and Joint Diseases, Shanghai Institute of Traumatology and Orthopaedics, Ruijin Hospital, Shanghai Jiao Tong University School of Medicine, Shanghai 200025, P. R. China

**Keywords:** tendon/ligament repair, biomaterials, electrospinning, hydrogel, tissue regeneration

## Abstract

With an increase in life expectancy and the popularity of high-intensity exercise, the frequency of tendon and ligament injuries has also increased. Owing to the specificity of its tissue, the rapid restoration of injured tendons and ligaments is challenging for treatment. This review summarizes the latest progress in cells, biomaterials, active molecules and construction technology in treating tendon/ligament injuries. The characteristics of supports made of different materials and the development and application of different manufacturing methods are discussed. The development of natural polymers, synthetic polymers and composite materials has boosted the use of scaffolds. In addition, the development of electrospinning and hydrogel technology has diversified the production and treatment of materials. First, this article briefly introduces the structure, function and biological characteristics of tendons/ligaments. Then, it summarizes the advantages and disadvantages of different materials, such as natural polymer scaffolds, synthetic polymer scaffolds, composite scaffolds and extracellular matrix (ECM)-derived biological scaffolds, in the application of tendon/ligament regeneration. We then discuss the latest applications of electrospun fiber scaffolds and hydrogels in regeneration engineering. Finally, we discuss the current problems and future directions in the development of biomaterials for restoring damaged tendons and ligaments.

## Graphical Abstract

**Figure:**
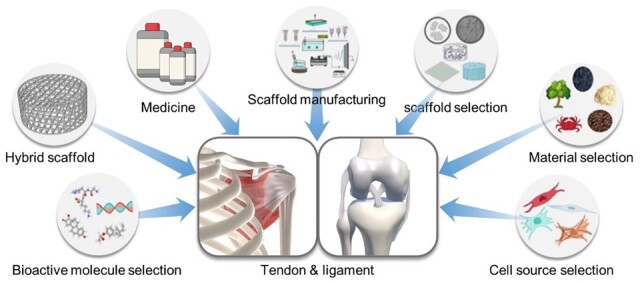

## Introduction

Common fibrous connective structures between bones and muscles include ligaments and tendons [1, 2]. They are crucial for preserving the structure and performing the musculoskeletal system functions because they stabilize forces, stabilize the bones, transmit mechanical forces and participate in bodily motions [3].

The body has numerous joint tendons and ligaments, including the anterior cruciate ligament (ACL), supraspinatus tendon (SST), rotator cuff tendon (RCT) and Achilles tendon (AT) [4–6]. The incidence of Achilles tendon (AT) rupture has significantly increased from 1.8 per 100 000 person-years in 2012 to 2.5 per 100 000 person-years in 2016 (*P* < 0.01), for an overall incidence of 2.1 per 100 000 person-years, making it one of the most prevalent tendon ruptures. The population incidence of quadriceps tendon rupture (QTR) was 0.48, and patellar tendon rupture (PTR) accounted for 13.5% of all knee injuries (QTR; 9% of knee injuries; the population incidence was 0.31) [7]. According to reports, the Western world—including the USA, Europe and Australia—sees the highest annual incidence of T/L ruptures and related repair procedures: RCT: 16–131 per 100 000 [8]; AT: 7–40 per 100 000 [9]; and ACL: 8–50 per 100 000 [10].

Although technology has advanced in recent years, the issue of rehabilitation of tendon injuries has not met everyone’s expectations and remains a serious clinical challenge [11]. The conservative clinical approach uses fixed casts and restricted movement orthoses, which require a long ongoing rehabilitation program to achieve functional recovery [12]. Standard surgical options for clinicians include filling a partial T/L defect with a graft or, if the defect is large, filling the graft with bone or muscle to completely replace the T/L [13]. However, these graft materials often differ significantly from the body’s tendon ligament properties in biocompatibility and mechanical strength [14, 15]. Therefore, there is an urgent clinical need to develop suitable synthetic graft biomaterials that can facilitate the repair and regeneration of damaged T/L while maintaining as many biomechanical properties as possible [16].

## Tendons and ligaments

### Main components, histology and morphology, and function of tendons and ligaments

From a microscopic perspective, triple helix type I collagen is an essential component of the fibers, which twist around each other to form bundles. The bundles accumulate by turning to create tendon units [17]. The tendon sheath surrounds the tendon bundle and includes the inner and outer cords [18].

**Figure 1.**
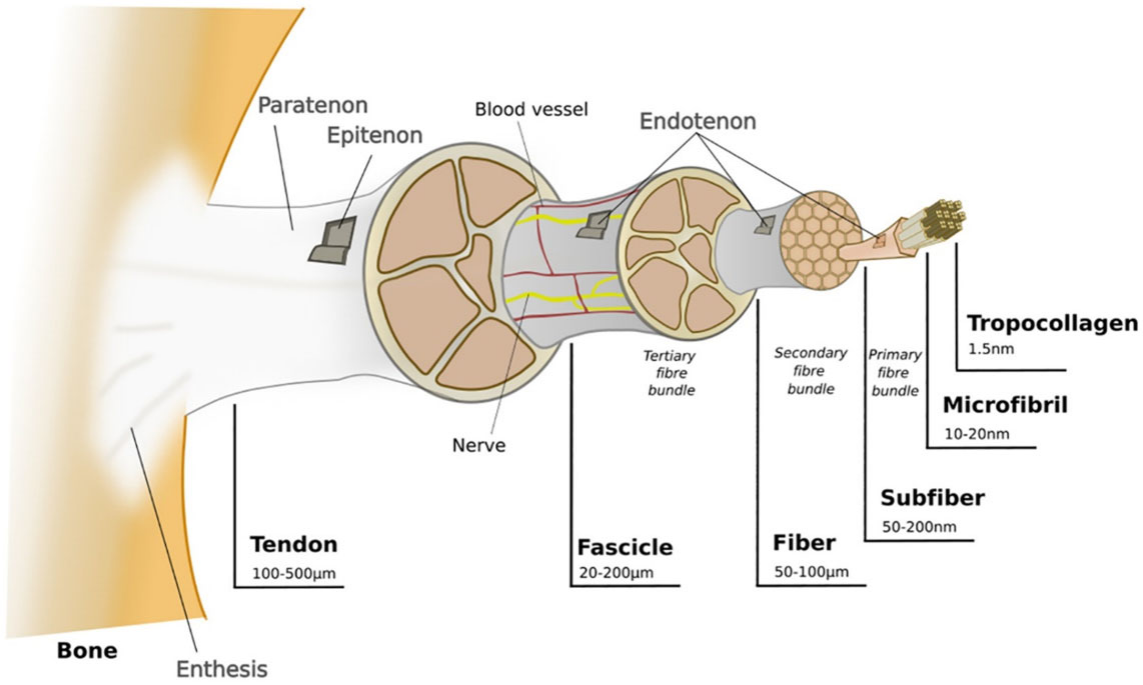
A tendon has many layers. Continuous stretching and contraction are due to the directional arrangement of collagen fibers, layered tissue (microfibrils, sub fibers, fibers and bundles), the composition of ECM and different membrane or sheath structures. Reproduced with permission [21]. Copyright 2021 Elsevier.

Tendons are mainly composed of highly aligned collagen-rich proteins and therefore have a higher tensile strength than the average tissue [19]. Tendons exist between muscle and bone. Their primary function is to transmit force and stabilize joints. Tendons resist tensile forces and are vital during human movement [20]. Ligaments contain many collagen fibers and elastic fibers (Fig. 1). The role of collagen fibers is to give ligament strength and stiffness. The elastic fibers allow the ligaments to stretch and extend under load [21]. Ligaments can strengthen joints, increase their stability during movement and prevent them from dislocating and causing injury [12]. Most fibers are aligned almost parallel to each other, so their force characteristics are usually such that they are subjected to loads in only one direction [22].

### Advances in repair processes and biomaterials for tendon and ligament rupture

If a tendon or ligament breaks, the repair process is relatively slow compared with other musculoskeletal tissues [23]. At the beginning of the rupture, inflammatory cells infiltrate the injury site, triggering the migration of tendon cells, accompanied by the proliferation of tendon cells and the production of type III collagen [24]. The remodeling stage begins 6 weeks later, the amount of type I collagen increases continuously, and the stress direction is consistent with that of tendon cells [25].

Complete rupture of the fibrous tissue usually takes 10 weeks to grow into scar-like tendon tissue, and the metabolic activity of the tendon cells and blood vessels gradually decreases [26, 27]. The repaired tendon is usually less intense than a healthy tendon due to a lack of mechanical stimulation, which is enhanced during the repair phase [21]. T/L ruptures have also proven to be the most difficult to treat due to their characteristics of poor blood supply to the tendon tissue leading to nutritional deficiencies [28].

Based on the current situation, the choice of synthetic or biomimetic materials made of polymers to mimic the structure and biological properties of tendon/ligament tissue for the repair of tendons and ligaments deserves further investigation [29]. Technologies such as electrostatic spinning and hydrogel microspheres, 3D printing and multifunctional coatings have been widely used in research and development toward the preparation of bionic structures that meet the requirements in terms of biocompatibility, biodegradability, mechanical properties, morphology, porosity, etc. [30]. Although there is still room for improvement in tissue organ bionics, there has been significant progress in the research and in the application of regenerative scaffolds for transplanted tissues, which is a massive advancement for tendon and ligament repair [31].

## Products of tendon and ligament reconstruction and repair

**Figure 2.**
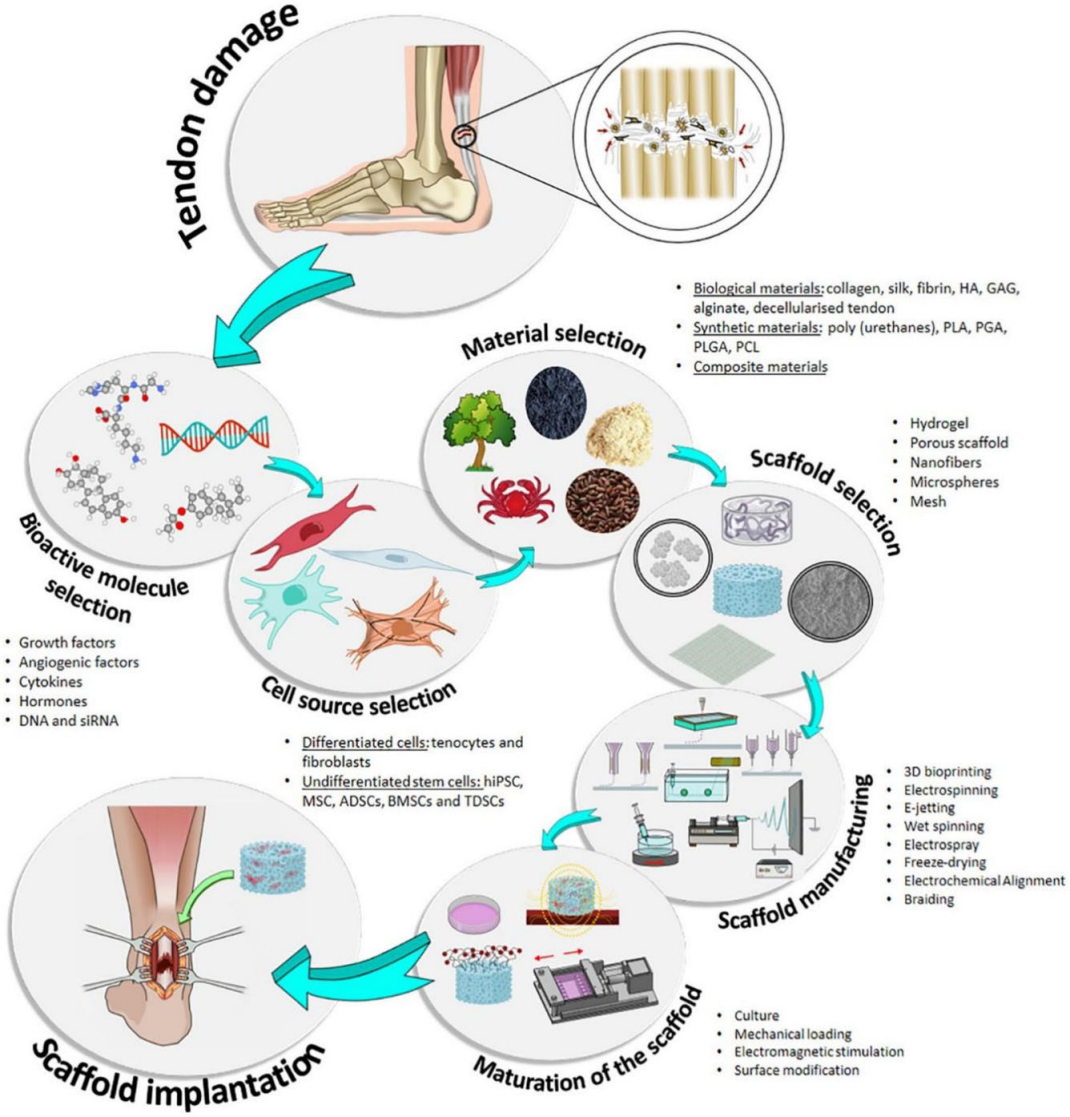
Biomaterials commonly used for T/L tissue regeneration. Reproduced with permission [21]. Copyright 2021 Elsevier.

Researchers often divide scaffolds into natural polymer (Fig. 2), synthetic polymer, composite and extracellular matrix (ECM)-derived biological scaffolds according to the properties of materials currently used in T/L injury [32].

### Natural polymer

Scaffolds made of natural polymers have unique advantages: better cell activity and adhesion than other materials and better biocompatibility and degradation rate. Therefore, this material has a bright future in tendon repair [33]. Natural materials commonly used for scaffold development include collagen, fibrin, silk, chitosan, hyaluronic acid and alginate [1, 34–37].

Collagen is the main component of the ECM in human tendons and ligaments; therefore, it is also the first natural polymer used to make scaffolds [38]. As collagen scaffolds have good binding sites for cells and growth factors, they support cell adhesion, migration, growth and differentiation [39]. Currently, the most commonly used collagen comes from animal tissues. It is necessary to remove antigens and pathogens through crosslinking to ensure that its immunogenicity meets the standard and improves its mechanical properties [40]. However, these processes reduce the material’s biomechanical strength. After physical or chemical crosslinking, collagen scaffolds lose the mechanical properties of natural collagen tissues, and their degradation rate, in the long run, is increased [41].

To overcome the problems of mechanical properties and degradation rate, researchers have attempted to inoculate autologous tendon cells on collagen scaffolds. Collagen scaffold-inoculated tendon cells were significantly closer to the natural tendon in terms of elongation and stiffness. However, this experiment was conducted only in an acute defect model. Tendons and ligament repairs are long-term processes. Whether it is still effective in a natural human environment remains to be determined [42]. Researchers have attempted to use new technologies to make better use of collagen. They creatively used counter-rotating extrusion technology to manufacture the aligned (CM_a_, orientation angle 0–15°) and randomly-oriented collagen membranes (CMr, orientation angle -60°-60°) from insoluble collagens. CM_a_ promotes tendon differentiation of rat bone marrow mesenchymal stem cells (rBMSCs) *in vitro* by inducing the shape of slender cells on the aligned fibers. CM_a_-BMSCs constructs can achieve tendon tissue healing by promoting tendon differentiation *in vivo* [43]. Puetzer *et al*. [44] offer a method for developing collagen scaffolds for tendon, ligament and meniscus tissues to reduce collagen degradation in musculoskeletal tissues and achieve better tensile properties. These structures hold considerable promise as functional musculoskeletal alternatives, and these models may be a promising tool for studying collagen fiber development, disease and injury *in vitro*.

Silk has good strength, toughness and an excellent degradation rate *in vivo* [45]. In vivo, the tensile strength of silk fiber can often maintain a good level for one year and degrade entirely within 2 years, allowing the mechanical load to be gradually transferred from the scaffold to the new ligament [46]. Structural scaffolds are an essential part of tissue regeneration. The study of silk fibroin (SF), a natural protein with outstanding mechanical properties, biodegradability, biocompatibility and bioresorbability has received significant attention over the years for tissue engineering (TE) applications [47]. Through various fabrication methods, SF can be transformed into films, mats, hydrogels and sponges, all of which can be dissolved into an aqueous solution. Multiple techniques can crosslink materials, such as spin coating, electrospinning, freeze-drying and physical and chemical approaches. The use of micropatterning and bioprinting techniques for fabricating SF-based scaffolds has been explored recently to facilitate the fabrication of more complex scaffolds [48]. By fabricating biphasic silk fibroin scaffolds, Font Tellado *et al*. [49] simulated the gradient in collagen molecule alignment at the interface. Researchers have discovered that scaffolds mimicking the native interface may be more effective in supporting tissue regeneration. Researchers constructed a new scaffold, which is a silk collagen sponge scaffold woven with ligament-derived stem/progenitor cells sheets (LSPCs). The growth of collagen fibers after transplantation was good. Silk collagen sponge scaffolds implanted with LSPCs have good effects on cell proliferation and differentiation, ligament fiber regeneration and ligament bone interface healing [50].

Alginate is a polyanion with high charge density and hydrophilicity [21]. The structure of the alginate scaffold is similar to that of the ECM, and the scaffold exhibits high biocompatibility [51]. Through physical, chemical crosslinking and gelation, it can be implanted into the injury site using a sponge or hydrogel [52]. Alginate scaffolds can combine different cell lines and bioactive molecules, such as fibroblasts or type I collagen, which can be accurately released by adjusting the type and cross-linking method [53]. The researchers confirmed the good cell proliferation activity of the alginate scaffold by assay and the sustained and stable release of TGF-β1 in the alginate scaffold by ELISA. This led to the conclusion that the continuous release of TGF-β1 from alginate scaffolds improves biomechanical and histological outcomes [54]. Despite its various advantages, compared to synthetic polymers, alginate also has a common disadvantage to natural polymers, namely a lack of mechanical properties [55].

Chitosan is a polysaccharide cationic polymer with good biocompatibility and hydrophobicity [56]. Chitosan can be processed into solid porous scaffolds that can be used for scaffolds and have excellent adhesion [57]. Willbold *et al*. [58] implanted Poly (ε-caprolactone) (PCL) scaffolds with chitosan polycaprolactone in rats and an infraspinatus tendon defect model. They found that this scaffold promoted vascularization near the wound, with histology showing that cell proliferation and differentiation increased significantly. Chitosan bridging polymer was coupled unilaterally to dissipative alginate acrylamide hydrogels to achieve the required tissue adhesion. For the treatment or prevention of tendon injuries, it was hypothesized that Janus Tough Adhesive (JTA) would simultaneously provide mechanical tissue integrity and controlled spatial and temporal drug delivery [59].

Hyaluronic acid (HA) is an anionic polysaccharide widely present in soft tissues and is responsible for maintaining normal ECM structure [60]. Because the degradation rate of HA is fast, combined with the fact that HA usually exists in the form of condensation, some crosslinking and chemical modification must be used to improve its mechanical strength and biodegradability [61]. Hyaluronic acid has shown promising efficacy and safety profiles in several clinical and preclinical studies, despite the absence of a consensus regarding a molecular weight classification. Hyaluronic acid has shown the potential to stimulate tendon healing in both *in vitro* and preclinical studies for its physical–chemical properties, such as biocompatibility, mucoadhesive, hygroscopicity and viscoelasticity. Hyaluronic acid has also been used to treat different tendinopathies in clinical trials with promising results [62]. Costa-Almeida *et al*. [60] combined negatively charged hyaluronic acid, alginate and chondroitin sulfate with positively charged chitosan to obtain multicomponent hydrogel fibers by photo-crosslinking and coagulation bath and cell encapsulation (Table 1), which were found to be suitable modulators of tendon cell bioactivity and have promising applications for tendon healing.

**Table 1.** Natural polymer materials for T/L reconstruction and repair

Material	Scaffold	Model	Result
Collagen protein	Autologous tendon cells were inoculated on collagen scaffolds	Sheep infraspinatus tendon defect model	The tensile strength of the reconstructed tendons (mean load to failure, 2516 N; SD, 407.5 N) was ∼84% of that of the native tendons (mean load to failure, 2995 N; SD, 223.1 N), the elongation and stiffness of tendon were significantly closer to that of the natural tendon. Fiber growth and collagen content were better [42].
Silk	Silk fiber collagen hydrogel scaffold	Rabbit anterior cruciate ligament defect model	Regenerated ACLs in the CSLS group tended to form larger collagen fibrils (47.5 ± 1.4 nm) than ACLs in the CS group (35.8 ± 1.8 nm), and the collagen fibers grew well [50].
Alginate	TGF was implanted into alginate scaffolds-βone	Rabbit supraspinatus tendon defect model	Exhibit a significantly heightened ultimate failure load (108.32 ± 32.48 N; *P* = 0.011), there were more collagen fiber bridging interfaces, and the area of new fibrocartilage formation was more evident [54].
Chitosan	Cs-g-PCL coated fiber scaffolds	Rat infraspinatus tendon defect model	The modulus of elasticity of the CS-g-PCL-patched group had a mean of 13.8 ± 5.4 N/mm^2^, which promoted the increase of vascularization near the wound. Histology showed that cell proliferation and differentiation also increased significantly [58].
Hyaluronic acid	Direct injection	Subacromial deltoid bursa in patients with supraspinatus tendon tear	Effectively relieved patients’ pain and improved shoulder function [63].

### Synthetic polymer scaffold

Polyesters, such as PCL, poly (l-lactic acid) and polylactic acid (PLA), are the main biodegradable scaffolds for T/L injury [64]. They have greater mechanical strength, better mechanical properties and better degradation rates *in vivo* than natural polymer materials [65–68]. Among these commonly used polymers, PCL has the slowest degradation rate, followed by PLA [69] and polyglycolide acid (PGA) [70]. In contrast, Poly (lactic-co-glycolic acid) (PLGA) has the fastest degradation rate [71].

PLA is a biodegradable and recyclable polyester produced from renewable raw materials [72]. However, synthetic polymers often have a dense structure and small pores, which are not conducive to cell proliferation and adhesion and hinder cell growth [73]. The studies found that after the ACL was replaced with the PLA-based implant, the failure forces of the knee joints were lower than those of intact joints, including the strain-at-failure rate. Due to the selected method of attaching autograft ends to the tibia and femur bone surfaces, the biomechanical parameters of the knee joint were substantially improved [74].

PLGA is a linear aliphatic polyester and a biodegradable, biocompatible polymer that can be used as a carrier to transfer growth factors or genes [75]. Jiang *et al*. [76] loaded plasmid DNA encoding fibroblast growth factor-2 into PLGA to evaluate its effects on human periodontal ligament cells *in vitro*. PLGA/pFGF-2 can promote periodontal ligament (PDL) regeneration. Han *et al*. [77] loaded bone morphogenetic protein 2 into PLGA and applied it to reconstruction after ACL rupture in rats. *In vitro* and *in vivo* experiments confirmed that BMP-2 and platelet-rich fibrin (PRF) could achieve sustained and stable release. The combination of this synthetic scaffold and growth factor effectively promotes the production of blood vessels and effectively controls inflammation and tendon–bone regeneration. A PLGA/wool keratin composite membrane loaded with basic fibroblast growth factor (bFGF) was prepared by Zhang *et al*. [78] through emulsion electrospinning. This composite membrane combines bFGF with dextran DEX to ensure that bFGF can be better encapsulated within the DEX and PLGA/wool keratin composite membranes. The electrospun emulsion fiber has a continuous core-shell structure that carries aqueous bFGF in the nuclear layer of the fiber and promotes adhesion, migration, proliferation, fiber growth and osteogenic differentiation of human periodontal membrane fibroblasts (hPLDFs).

### Hybrid scaffold

In earlier years, it was common to use only one material to create scaffolds [79]. However, clinical use and further research have highlighted many problems with this method, including poor mechanical strength and fast degradation rate of natural polymer scaffolds [80]. Synthetic polymer scaffolds have insufficient biological activity, making it difficult to promote cell adhesion and proliferation and inevitably produce acidic degradation products during degradation *in vivo* [81]. As a result, researchers began to consider the advantages of combining synthetic and natural polymer scaffolds to make up for each other’s defects, thereby achieving optimal biological conditions [82]. Therefore, composites made by organically combining several materials have significantly improved biocompatibility, mechanical strength and biodegradability [83]. Liu *et al*. [84] deposited polyelectrolyte multilayer films (PEM) with poly-l-lysine (PLL)/ hyaluronic acid (HA) on the scaffold which is fabricated with flexible and elastic poly(l-lactide-co-caprolactone) (PLCL 85/15). After PEM modification, medullary mesenchymal stem cells (BM-MSC) showed good metabolic activity on the scaffold and type I collagen. Type III collagen secreted by mesenchymal stem cells increased significantly, with good mechanical properties and biocompatibility.

Compared to traditional composites, nanocomposites exhibit unique properties. Nanoparticles have a higher surface-area-to-volume ratio, better ductility than general materials and good mechanical strength and biodegradation rate [85]. Green [86] attempted to combine nanomaterials with collagen. Collagen/carbon nanofibers were prepared with 0.5 and 5 wt% filler loading, using 1× Phosphate buffered saline (PBS) diluted glutaraldehyde (GA) solution. Tensile tests were then carried out under dry and wet conditions, and their mechanical properties were found to be equivalent to those of natural collagen fibers. Tomas [87] decorated magnetic nanoparticles based on cellulose nano scaffolds to make the scaffolds magnetic responsive, promoted the growth of human adipose stem cells (HASCs) and tendon differentiation through mechanical sensing mechanisms *in vivo* and *in vitro* and found that pro-inflammatory markers were downregulated under magnetic conditions.

The regeneration of tendon and ligament tissues must be taken into account the problem of the interface. The regeneration of the interface is usually tendon–bone interface regeneration or muscle–tendon interface regeneration [88]. Considering the different physical properties, cells and growth factors of different interfaces, we should consider more composite scaffolds made of different materials [89]. In the tendon–bone interface, Zhang *et al*. [90] combined the degradable scaffold with the non-degradable scaffold to create a new scaffold similar to the ‘Swiss roll’ structure, decorated the bone morphogenetic protein 7 (BMP-7) on the PCL nanofiber membrane. They then rolled the nanofiber membrane and polyethylene terephthalate (PET) mesh fabric into the desired ‘Swiss roll’ structure. In *in vitro* experiments, the new tissue covering the surface of the BMP-7/PCL/PET mixed ligament was similar to the natural ACL ligament tissue. The gap between the main bone and the graft narrowed, many new bones were found, and many new blood vessels were formed at the interface.

### Natural ECM derivative scaffold

Different ECM-derived materials have been obtained from several different organs, such as the common skin, bladder, colon, small intestinal submucosa and esophagus [91, 92]. ECM-derived materials can promote macrophage polarization, and the mechanism of polarization promotion of ECM-derived materials from different sources varies [93]. For example, the skin induced M1 polarization of macrophages *in vitro*, wherein the specific mechanism involved the upregulation of iNOS. Several other common ECM-derived materials have been shown to induce macrophage M2 polarization *in vitro*, and the induction process is similar to that of IL-4 [94]. Acellular biological scaffolds have been widely studied recently and have wide application prospects for tendon and ligament healing [95]. On the premise that the mechanical properties and ECM structure are well preserved, the acellular biological scaffold also has a better solution to the immunogenicity of transplantation since this scaffold removes the cellular components of tissue [96]. Acellular biological scaffolds have a good cell structure. Still, compared with synthetic polymer scaffolds, acellular biological scaffolds are more difficult to obtain raw materials, which is an obstacle in practical clinical applications [97]. Thirty individual small intestine submucosa (SIS) sheets were laminated and then grafted with SIS patches to repair tendon defects. The enhanced, low-immunogenicity SIS patches were biomechanically and structurally equivalent to their broad fascia. Although the results are good in short-term acute tendon defects, the results for chronic defects are yet to be proven [98]. The amniotic membrane and chorionic layer were washed, laminated and dehydrated to prepare the micronized dehydrated human amniotic membrane/chorionic membrane (μdHACM). The double-layer tissue was ground at a low temperature, and the particles were collected and applied to the tendon cell inflammation model *in vitro*. μdHACM treatment was found to reverse the pro-inflammatory-induced increase in type 3 collagen expression and decrease the IL1β-induced type 1 and type 3 collagen levels. Researchers have also found that μdHACM can promote angiogenesis [99]. The decellularized biological scaffold can also be an effective alternative for future tendon ligament injuries after ensuring mechanical properties and avoiding rejection *in vivo* [100].

### Products for clinical trials

Surgical reconstruction of the ACL may involve artificial ligaments as either an augmentation to autologous grafts, allografts or a complete replacement. However, in early clinical trials, most artificial, non-degradable devices have not proven satisfactory over time. The Gore-Tex polytetrafluoroethylene (PTFE) ligament was developed as a prosthetic ligament but has also been used as an augmentation device. However, clinical studies with long follow-up times have shown a high incidence of complications [101]. There has been some evidence of a higher rate of complications with an additional poly(urethane urea) degradable augmentation device in a single-bundle bone-patellar tendon-bone (BPTB) ACL reconstruction compared with non-augmentation in short interim or long-term studies. As a result of the augmentation of the autograft, 10 patients had lengthy surgical procedures and later explanations of the device, six of these because of an insufficient screw fixation to the femur and four due to swelling [102].

In several clinical studies in recent years, the choice of implants for tendon ligament injuries has increased as the range of materials and synthetic techniques have been improved. With this has come a degree of improvement in the effectiveness of the implants found in clinical trials. Bridge-enhanced ACL repair (BEAR) may be a viable alternative to ACL reconstruction (ACLR) for complete mid-segment ACL tears. At two years postoperatively, anteroposterior (AP) knee laxity and patient-reported outcomes were similar between ACL repair using the BEAR implant and ACLR [103]. This procedure has inherent advantages, including lacking an autograft requirement and less risk of osteoarthritis following the process. This study suggests that ACLR repair using the BEAR implant is a safe and promising technique that warrants further investigation.

As well as inducing new tendinous tissue to grow on the bursal surface of the supraspinatus tendon, the collagen implant also improved the quality (based on MRI) of the native tendon. Following surgery, the implant-generated host tissue matured over time and remained stable at 12 months, and the average increase in tendon thickness was 2.2 mm at three months. Previous studies on sheep and humans have shown similar results. Recent studies have confirmed that these implant-generated host tissues can rapidly mature into tendon-like tissue following rotator cuff repair [104, 105].

A potential disadvantage of tendon/ligament repair in clinical trials is that this method requires tearing down the intact tendon/ligament tissue, which may result in a length-tension mismatch in the repaired cuff and alter the normal orientation, and shape [106]. As a result, it may be necessary to immobilize a surgically repaired shoulder for six weeks after the operation before gradually progressing to active motion and strengthening over a six-month rehabilitation period [107]. As a result of the bioreductive implant, patients with intermediate- to high-grade partial-thickness lesions might have experienced a more rapid recovery than expected after postoperative rehabilitation. To prevent increased strain on the tendon, physicians and their patients are interested in surgical procedures that preserve the native cuff anatomy while biologically augmenting the degenerative tissue.

As a result of the degradable augmentation device, tissue can be ingrown into the graft and tissue tensioned, providing natural biomechanical stimulation to the graft [108]. As a result of necrosis and resorption and an unprotected period of regeneration, the autograft loses strength and elasticity after surgery. A degradable augmentation device shares the mechanical load with the biological graft by gradually increasing the stress on the autograft. Future studies must investigate the stress shield distribution from the degradable augmentation device to the autograft.

## Electrospun fiber material for tendon repair

The simulated natural ECM produced by electrospinning provides sufficient conditions for cell anchoring, adhesion, proliferation, migration and differentiation [109]. Researchers have recently combined electrospun nanofibers with emerging materials, such as minerals, metals, growth factors, stem cells, drugs and nanoparticles, to enhance further their physical and chemical properties and biological activities [110]. The structure and porosity of the scaffolds should promote cell activities and the formation of new tissues [111].

**Figure 3.**
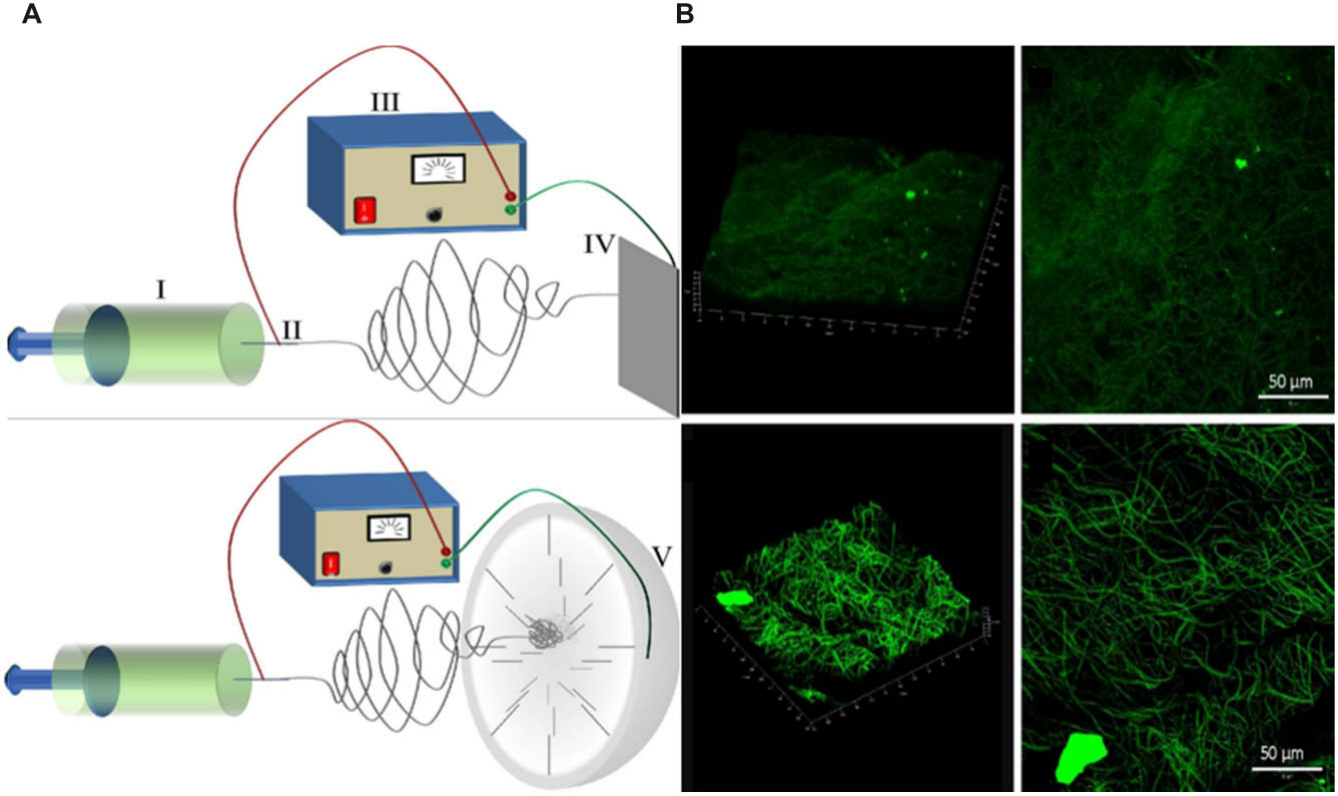
(**A**) Traditional flat-plate collector, spherical disk and metal array collector. (**B**) In three-dimensional rendering and two-dimensional projection, a cotton ball-like ePCL scaffold has a loosely packed network structure [116]. Copyright 2011 Elsevier.

An essential disadvantage of electrospun fiber scaffolds is their low porosity and unsuitability for implant cell infiltration [112]. In particular, the pore size left for cells was smaller after the scaffolds were knitted, braided, twisted woven fibers and 3D printed. Researchers have attempted to limit the melting between fibers in the electrospinning process to separate the pore and fiber diameters in the electrospun support [113]. Olvera *et al*. [114] designed a porous three-dimensional (3D) microfiber scaffold by changing the rotation speed of the collection mandrel. The fibers had a porosity of 95%, making it easier to bend the electrospun sheet. Their mechanical properties were similar to those of the natural anterior cruciate ligament. Higher porosity also allows mesenchymal stem cells to be better immersed in implants. To enhance cell attachment, spreading and proliferation while increasing the size of the fibers, researchers have shortened the distance between the nozzle and the collector, electrospun the HA/collagen mixture with N,N-dimethyl formamide (DMF)/NaOH as the solvent, and produced specific pore sizes on the whole scaffold through chemical crosslinking and immersion of sodium chloride particles during electrospinning. Cell adhesion, growth and collagen content were enhanced in an *in vitro* model of bovine chondrocytes. However, they cannot provide uniform morphology and stability [115]. Blakeney *et al*. [116] developed a 3D electrospun scaffold with a shape similar to that of a cotton ball, composed of loose and porous nanofibers (Fig. 3A). In contrast to the traditional flat-plate collector, the non-conductive spherical disk of the embedded metal probe creates a Focused, Low density, Uncompressed nanoFiber (FLUF) mesh support (Fig. 3B). The cotton ball support comprises electrospun nanofibers with similar diameters, larger pores and a lower structural thickness.

Tendons and ligaments have an anisotropic layered structure, and how to better model their structure and avoid inter-fiber wear is of great importance to ensure the long-term function of the graft [117]. A hybrid nanofibrous composite is presented here to imitate these characteristics. PLCL and gelatin nanofibers are electrospun sequentially onto PET fibers to fabricate nanofiber-reinforced yarns for improved durability and biocompatibility [118]. They compared different manufacturing methods and determined that the knitted structure could simulate anisotropic mechanical properties, even, more potent than natural ligaments. Also, *in vivo*, tendon regeneration is promoted by the knitted nanofibrous composites on which ligaments are formed. A significant amount of tendon-associated ECM proteins is deposited after optimized anisotropic hybrid nanofibrous composites for tendon repair [119].

Electrospun fibers are often mechanically inadequate for tendon applications. It is therefore a new idea to design a reinforced fiber and improve the electrospinning method to enhance the deposition and integration of fibres when electrospinning fibers are fabricated. Li *et al*. [120] have reported a strategy based on a rough polymer surface reinforcement as part of the surface fiber deposition and integration during electrospinning. An open-pored polymer surface significantly improved fiber deposition when added to polymer surfaces. The fabricated fiber topography had improved surface hydrophilicity, a higher crystallinity and improved mechanical properties such as maximum force and maximum extension following elastic and plastic deformations. In constructing tendon cell interconnections with tendon stem cells (TSCs), the fiber topography suggested good cell compatibility and the ability to support F-actin cytoskeleton expression. The results of this study could provide insights into how mechanically enhanced fiber topography can be designed to regenerate tendon tissues. The mechanical properties have been improved, but the tendon adhesions and inflammation problems still need further improvement, and for these two persistent problems, Pien *et al*. [121] offers a new idea. To fabricate tubular repair constructs, naproxen and hyaluronic acid (i.e. anti-inflammatory and anti-adhesion compounds) were electrospun into the acrylate-endcapped urethane-based polymer (AUP) material and mechanical reinforcement with a tubular braid. Researchers tested the developed tendon repair constructs using *ex vivo* sheep tendons. They found that they possessed the mechanical properties for tendon repair (i.e. minimum ultimate stress of 4 MPa), with a maximum pressure of 6.4 ± 0.6 MPa. In addition to the innovation of the electrostatic spinning material itself, the way it is secured at the wound site has an equally profound impact on wound healing. As electrospun nanofibers can slip under external forces, thus hindering the proliferation and differentiation of migrating stem cells, Wang offers a new way of thinking about how to anchor them more firmly and improve their ability to promote tissue regeneration [122]. Poly(ester-urethane) urea and gelatin were electrospun and double crosslinked by a multi-bonding network densification strategy to create nanofiber scaffolds with formed joints to mimic the natural microstructure of tendon-to-bone insertion. However, a crimped nanofiber scaffold (CNS) has been shown to induce chondrogenic differentiation and bionic tensile stress, making it a credible platform for *in vivo* experiments [123].

**Figure 4.**
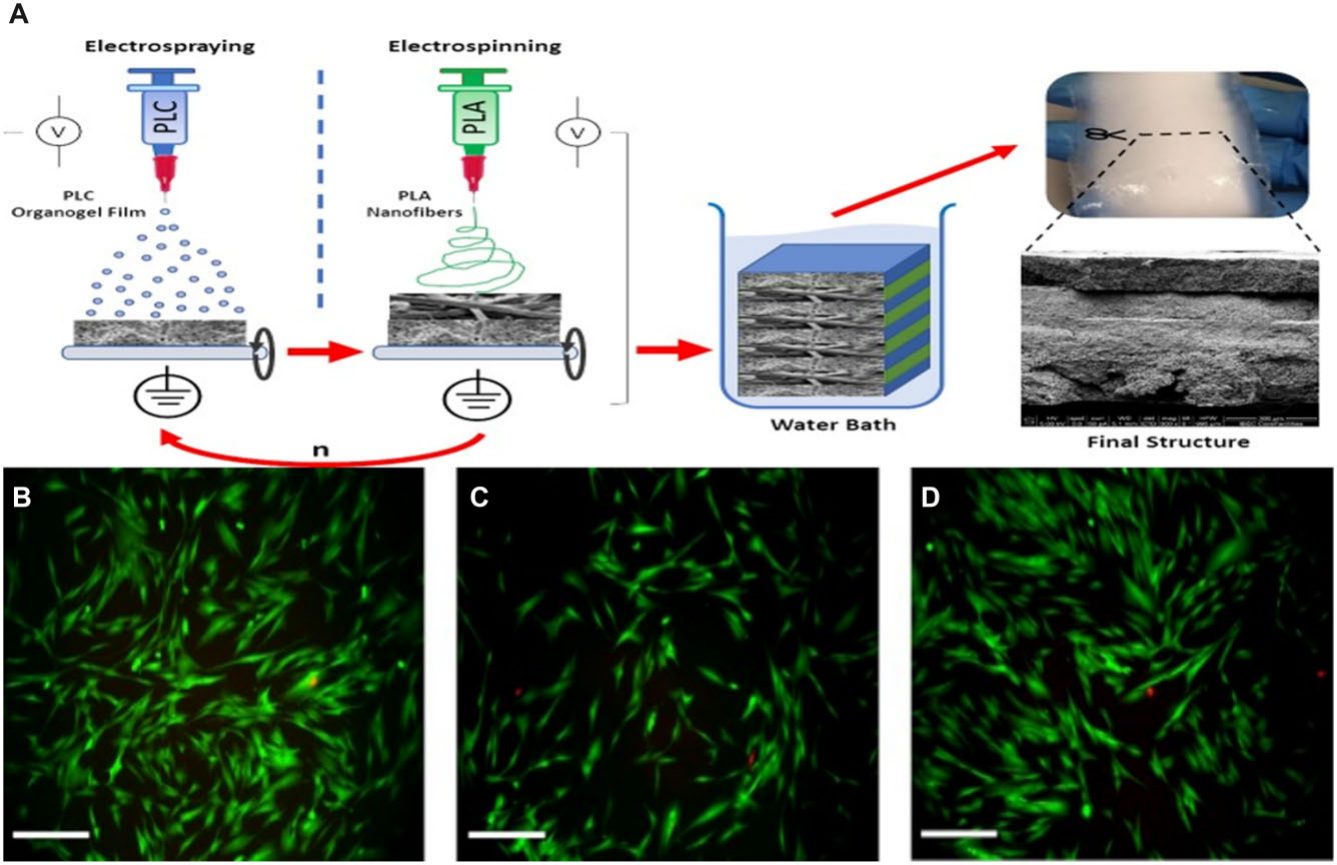
(**A**) Preparation of RCA patch, combining PLC thin film produced by electrospray and PLA nanofiber produced by electrospinning and immersion in water bath precipitation polymer to obtain the final product. (**B**) In the control group, cell metabolism and growth factor activity were detected alive and dead. (**C**) Cell metabolism and growth factor activity were detected in 4L35/40 medium. (**D**) Cell metabolism and growth factor activity were detected in the F4L35/40 medium. Reproduced with permission [124]. Copyright 2019 Rey-Vinolas *et al*.

The integration of electrospinning with other technologies compensates for the technical shortcomings of both sides and gives full reign to the advantages of both technologies (Fig. 4) [124]. For example, electrostatic spinning combined with 3D technology, electrostatic spinning technology combined with electrospray, rotary jet spinning (RJS) and wet electrostatic spinning (WES) can all complement each other’s strengths. PCL–poly (glycerol sebacate) (PGS) blends can be made into scaffolds by electrostatic spinning techniques, and 3D printing techniques are also widely used. Touré *et al*. [125] innovatively electrospun fiber mats of PCL and PGS directly onto one side of a 3D printed grid of PCL–PGS blends containing bioactive glass, and the scaffolds exhibited good three-dimensional porosity for cell growth and infiltration. The scaffold showed good three-dimensional porosity for cell growth and infiltration, superior mechanical properties and degradation rates. The combination of electrostatic spinning and electrospraying to produce poly-L-lactic-co-ε-caprolactone (PLC) films in the form of organogels and PLA nanofibers produced by electrostatic spinning to make rotator cuff reinforced patches resulted in a combination of the two techniques to form a structure with good mechanical properties and good biocompatibility [126].

**Figure 5.**
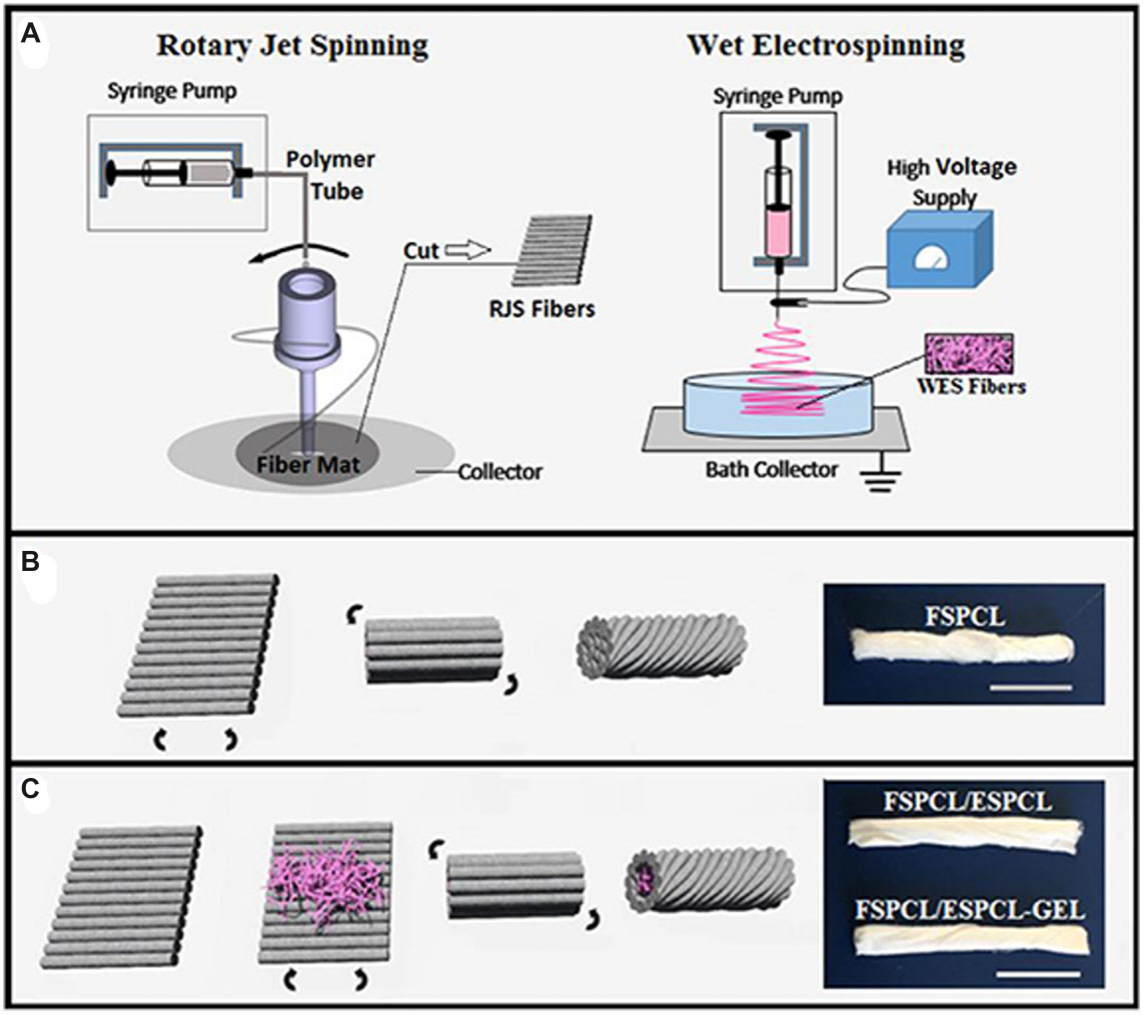
Fabrication of biphasic fiber support. (**a**) three stent forms (**b**) FSPCL (**c**) FSPCL/ESPCL and FSPCL/ESPCL-gel ratio 3:1 stent. Reproduced with permission [127]. Copyright 2019 IOP Science.

Guner *et al*. [127] assembled fiber pads produced by rotary jet spinning (RJS) and wet electrospinning (WES) into biphasic fiber support. The shell of the biphasic support is composed of aligned PCL fibers to ensure a better mechanical property and strength (Fig. 5a). The support core was composed of PCL or PCL/gelatin fibers, and their arrangement was random, providing a bionic structure suitable for cell adhesion (Fig. 5b). Subsequent *in vitro* studies showed that the core fibers were randomly arranged in the aligned PCL fiber shell of the biphasic scaffold, which increased the initial adhesion, proliferation and differentiation of the mouse fibroblast cell line (Fig. 5c). The aligned arrangement of PCL fibers can promote the elongation of aligned fibers, thereby improving tendon tissue healing by guiding cell proliferation and ECM deposition.

## Hydrogel materials for repairing tendons and ligaments

Hydrogels are 3D network structures that are hydrophilic, similar to the ECM structure. Hydrogels can be combined with growth factors, cells and drugs and have been widely used for drug delivery, wound treatment and cell culture [128]. Although hydrogels have many advantages, their use remains outside the realm of clinical application, including in T/L tissue, due to several disadvantages, including poor mechanical properties, fragile stress, stiffness and natural tendons and ligaments [129]. To bring the hydrogel closer to the tendon/ligament in terms of structure and properties, Park [130] proposes a strong, tough, hierarchical hydrogel that mimics tendon function at multiscale levels. A double-network isotropic hydrogel is transformed into an anisotropic hydrogel via stretching, solvent exchange and subsequent crosslinking via ionic forces. Based on the degree of stretching, anisotropic hydrogels display high strength and toughness that fluctuate over a wide range (1.2–3.3 MPa of strength). In addition, anisotropic hydrogel strands are braided into a rope to form a hierarchical architecture that exhibits an improved mechanical performance (4.7 MPa of strength in a four-strand hydrogel rope) compared with separated hydrogel strands (2.3 MPa of strength). Anisotropic ligaments consist of bundles of collagen fibers with a high degree of mechanical strength and directional movement when stimulated by external stimuli such as temperature and strain [131, 132]. Hydrogels must be designed to synergize composition and micro/macro anisotropic structures to achieve truly biological simulations. Hydrogels can be constructed with anisotropic structures using various techniques, including supramolecular self-assembly, electric, magnetic and force fields, as well as directional freezing [60, 133–136]. Anisotropy of hydrogel fibers is enhanced through the microstructural alignment of clays and polymers during stretching. By varying stretching ratios, the fibrous gel bands retain their anisotropy, giving them robust mechanical properties and a fast anisotropic shape deformation response to external thermal stimulation. This method can be expanded to manufacture a variety of anisotropic actuators by further designing and fabricating bilayer fibrous gel tapes. An excellent platform for developing next-generation soft materials for bionic tissues has been developed through this study [137]. Using the gel aspiration-ejection (GAE) method, collagen hydrogels are rapidly densified and remodeled into aligned dense collagen (ADC) systems. The removal of the casting fluid increases collagen fibrillar density (CFD, or collagen content) [138], while the applied pressure differential aligns the resulting hydrogel. ADCs provide a microenvironment similar in composition and structure to tissue, with highly aligned collagen, with regenerative potential in terms of short-term mechanically activated tendon differentiation [139].

In addition to enhancing the mechanical properties and structure of the hydrogel, the prevention of adhesions in the peri-tendon tissue also has a profound effect on injury repair. Dang *et al*. [140] developed a unique hydrogel using Skin Secretions of Andrias Davidianus (SSAD), which is biologically active, adhesive and possesses controllable microstructures. SSAD-derived hydrogel with double layers demonstrated strong adhesiveness *in vivo* and could repair ruptured Achilles tendons in rats without suturing. SSAD-derived hydrogels are their antioxidant, and antibacterial properties are further beneficial, which promote peritendinous adhesion reduction. Postoperative tendon sheath adhesion is an essential factor that affects the postoperative rehabilitation of tendon and ligament injuries [141]. Many patients experience poor functional recovery due to adhesions. Researchers have used many methods to reduce this adverse adhesion, including minimally invasive sutures, drug treatment and postoperative rehabilitation; however, the problem of postoperative tendon sheath adhesion remains outside their grasp [142]. Imere *et al*. [143] assembled a PCL self-assembled peptide hydrogel using B synovial cells. The synovial cells encapsulated in hydrogels promote hyaluronic acid production, restore tendon lubrication and help synovial sheath regeneration to prevent tendon sheath postoperative adhesion. Promoting tendon ligament soft tissue regeneration represents a new approach.

In addition to improving post-operative tissue adhesions, enlargement of the bone tunnel is a recurring phenomenon after tendon/ligament graft implantation, affecting the long-term fixation of the implant [144]. Micromotion of the graft within the tunnel, vibration or defective fixation techniques are the mechanical causes of this condition [145, 146]. Intra-articular and synovial fluid accumulation between the tendon/ligament and the bone wall may also lead to local osteolysis and tunnel enlargement, the so-called ‘synovial bath effect’ [147]. The construction of bionic composite tubular grafts (CTG) made from horseradish peroxidase (HRP)-cross-linked serine protein (SF) hydrogels containing ZnSr-doped β-tricalcium phosphate (ZnSr-β-TCP) particles as promising bone tunnel fillers for ACL graft (ACLG) implantation can improve the fixation of ACLG and facilitate tendon/ligament repair of tendon/ligament injuries [148].

Reliable mechanical strength, tendon-like structure and controlled drug release rates are important for wound recovery. In contrast, most hydrogels release drugs abruptly and intermittently, requiring sutures or penetrating cells to fuse with the surrounding tissue. In contrast, Benjamin R Freedman reported a hydrogel with a tough matrix on one side and a chitosan surface on the other, a hydrogel with a ‘Janus’ surface and sustained drug release, which acts as a high volume drug reservoir better supported by tendon gliding and adhesion. How effectively hydrogels release drugs, cells and biological factors is critical to wound healing, Decellularized tendon ECM has been widely studied as a biological scaffold [149]. However, the effect of acellular tendon hydrogels on stem cell behavior is minimal. Ning *et al*. [150] produced a new acellular tendon hydrogel (T-gel) using *Macaca mulatta*. The acellular tendon hydrogel affected the differentiation, proliferation and tendon regeneration of tendon-derived stem cells (mTDSCs). Compared with collagen gel (C-gel), acellular tendon hydrogel has a better porosity. The combination of hydrogel and pulsed electromagnetic field (PEMF) accelerates the release of drugs and relieves pain and tissue swelling often associated with tendon tissue injuries [151]. Wang *et al*. [152] combined celecoxib with pulsed electromagnetic fields (PEMF) and hydrogels to prepare a magnetic response hydrogel dressing containing celecoxib. In a rat Achilles tendon rupture model, Fe_3_O_4_ nanoparticles accelerated drug release and promoted M2 macrophage polarization at the lesion site, effectively alleviating inflammatory reactions and relieving pain in patients.

## Biological factors for repairing tendons and ligaments

Traditionally, repairing and healing injured and diseased tendons have been fraught with concern and difficulty, often leading to less satisfactory results [153]. Recent research on growth factors has opened up new treatment options for tendon injuries and diseases [154]. FGFs promote the development of tendons and muscles in tissues and organs, so it is hoped that research into utilizing their biological effects will result in new therapeutic methods [155]. However, vascular endothelial growth factor (VEGF) appears to adversely affect tendon healing in experiments because of its angiogenic activity and stimulatory effect on matrix metalloproteinases, which are highly expressed in various ruptured tendons [156].

The abnormal growth of blood vessels and the growth of bone tissue into the injured site are the main factors leading to poor healing of the tendon–bone interface and bone tunnel surface [157]. However, with advances in research, exogenous biological factors and cells have increasingly received more attention and are increasingly used in clinical settings [158]. Biological factors can interact with cells and regulate cell activity [159].

IGF-1 is highly expressed in the early stages of inflammation and promotes collagen production by tendon fibroblasts [160]. Muench *et al*. [161] applied insulin or IGF-1 to the tissue of the acromial bursa and then observed the migrated subacromial bursa tissue (SBT)-derived cells with a fluorescence microscope. Insulin and IGF-1 inhibited cell proliferation at the initial stage, subsequently promoting cell proliferation, differentiation and migration after 96 h.

**Figure 6.**
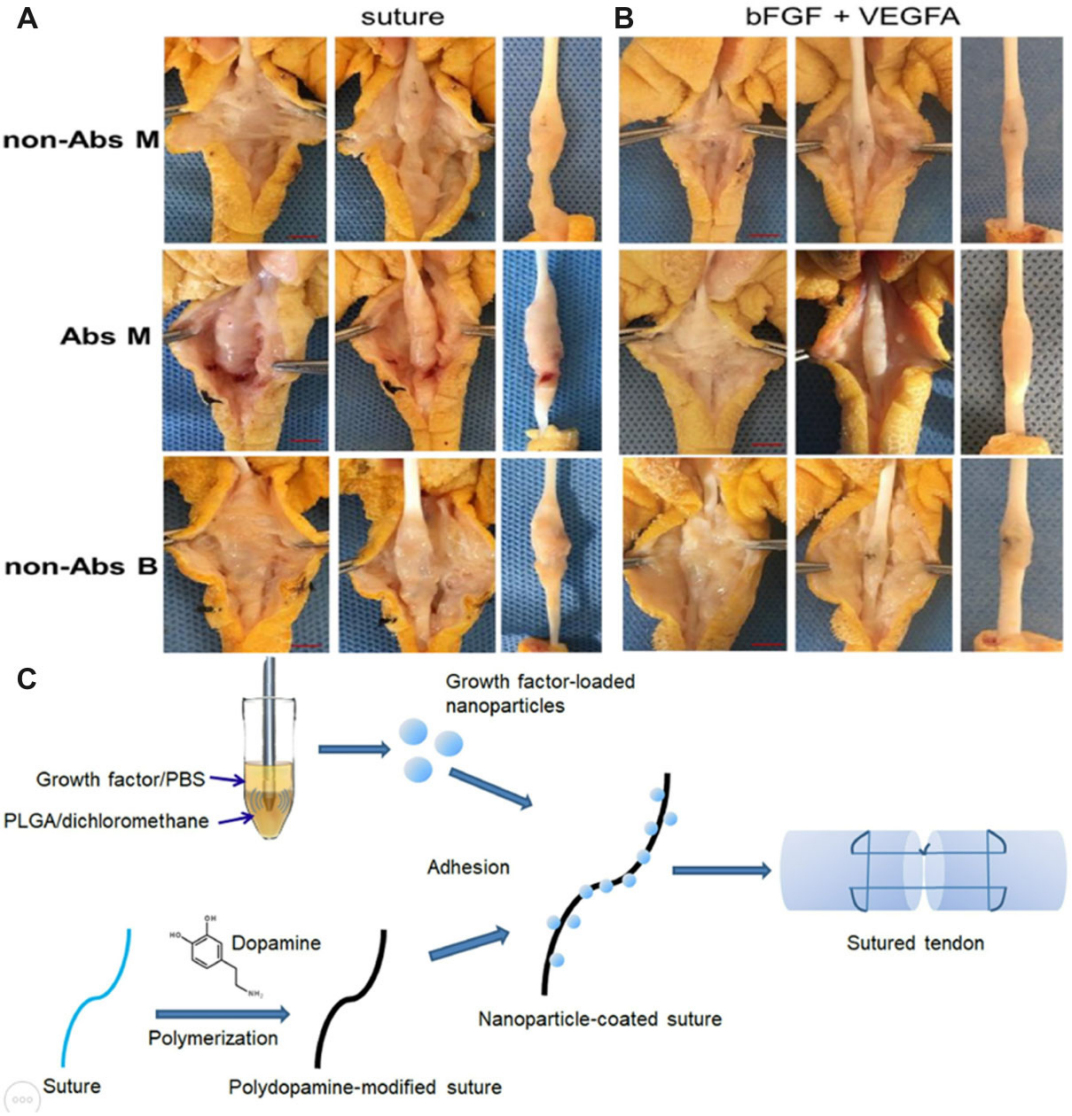
(**A**) Effect of ordinary suture on repairing tendon adhesion. (**B**) Effect of nanoparticle-coated suture loaded with bFGF + VEGFA on repairing tendon adhesion. (**C**) Process diagram of preparing nanoparticle-coated suture loaded with growth factor. Reproduced with permission [166]. Copyright 2021 Elsevier.

Essential fibroblast growth factor (bFGF) and vascular endothelial growth factor A (VEGFA) have been found in basic research to significantly stimulate cell proliferation and differentiation, fiber growth and collagen production [162, 163]. These growth factors effectively improve tendon healing. The sustained and stable release of growth factors is the key to promoting the healing of injured tendons [164]. Although many researchers have tried different methods, the effect remains poor. Surgical sutures are common in surgery and must come into contact with the damaged tissue [165]. With the successful loading of growth factors on sutures in recent years, Zhou *et al*. [166] attempted to evenly adhere PLGA nanoparticles encapsulated with bFGF and VEGFA to the suture surface (Fig. 6A), effectively transporting growth factors to the tissue and controlling the release of growth factors. In the chicken flexor tendon injury model and rat Achilles tendon injury model (Fig. 6B), it was found that nanoparticle-coated sutures containing bFGF and VEGFA improved the adhesion of tendons significantly, improved the sliding function of tendons, and further promoted tendon healing (Fig. 6C). This growth factor delivery system has broad prospects for repairing tendon injuries and fractures.

Specific growth factors can promote the differentiation of adipose-derived stem cells into various mesodermal cells, such as tendon cells [167]. The transected tendon was treated with adipose stem cells (ADSCs) cultured with growth differentiation factor 5 (GDF5) and platelet-derived growth factor (PDGF). As a result, the tendon fiber and blood vessels grew well, promoting the tendon tissue’s healing [168].

The rotator cuff tear injury prevalence in humans is increasing annually [169]. Orthopedic doctors’ difficulty in dealing with this type of injury lies in the remodeling of the tendon–bone interface. Platelet-rich plasma (PRP) contains a variety of growth factors, the most abundant of which are transforming growth factor-β1 (TGF-β1), VEGF and PDGF [170]. These growth factors can regulate the recovery from inflammation and promote cell proliferation and differentiation [171]. Graphene oxide (GO) is a high-tech material with excellent performance. It can be used as a carrier for growth factors and drugs to improve drug release. Bao *et al*. [172] developed a GO/PRP gel to treat rotator cuff tears by combining PRP with GO. The gel had good biocompatibility, and the structure of the newly formed tendon-bone interface (TBI) tissue was more similar to the structure of the natural tendon–bone junction. It had better mechanical properties, and the gel promoted the proliferation, osteogenesis and cartilage differentiation of BMSCs. Thus, they have good application prospects. At present, the most difficult problem with the action of growth factors at sites of injury is the difficulty associated with achieving their sustained and controllable release and the control of the inflammatory response after the stent enters the body [173]. The combination of biomimetic nanoparticles (NPS) and scaffolds represents a new approach to promoting the sustained and effective release of drugs and growth factors [174].

## Cells that repair tendons and ligaments

**Figure 7.**
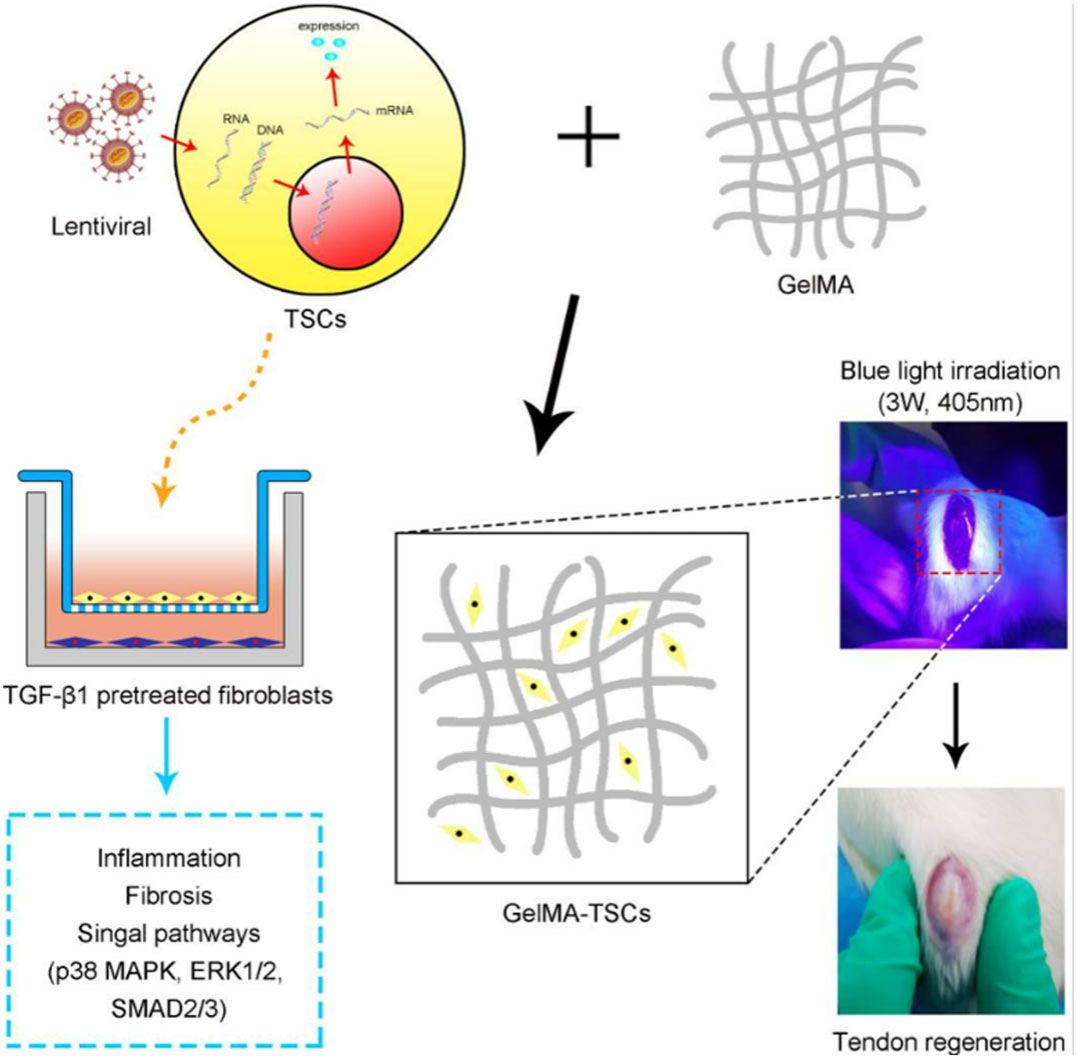
Tendon stem cells (TSCs) expressing hepatocyte growth factor (HGF) can stimulate a more orderly arrangement of collagen fibers and lower COLIII α-SMA, TGF-β1 levels and fibronectin, promoting tendon healing. Reproduced with permission [180]. Copyright 2021 Zhang, Liu, Shi, Zhang, Lu, Yang, Cui and Li.

Cell therapy involves injecting cells from other body parts or allogeneic sources into the injured site [175]. Currently, research in this field focuses on differentiated cells (tendon cells and fibroblasts) and stem cells (Table 2), the main cells constituting the ECM [176]. Tendon cells and fibroblasts can produce rich growth factors to promote the healing of injured sites, and they are also the most common cells in tendon tissue [177]. Stem cells, such as bone marrow stem cells, mesenchymal stem cells and induced pluripotent stem cells, have different regeneration abilities for other tissues and are applied to various tissues. After differentiation, these stem cells can produce tendon cells or fibroblasts. Stem cell therapy has special precautions: tendon cells and fibroblasts will not produce teratomas, and transplanting stem cells and other cells may lead to tumors [178]. The combination of cells, materials and growth factors can effectively control the location and degradation of cells and play a role in the damaged part for a long time without consuming it in other places to strengthen the effect of cell therapy [179]. The key for stem cells to improve tendon and ligament healing lies in the role of exosomes and secreted growth factors. The paracrine mechanism is critical for stem cells to promote tissue healing, and the hepatocyte growth factor (HGF) is an essential nutritional factor with paracrine activity, regulating inflammation and inhibiting fibrosis. Zhang *et al*. [180] transfected TSCs with HGF and found those collagen fibers arranged better (Fig. 7), reduced fibrosis, mild inflammatory reaction and promoted tendon healing.

**Table 2.** Cell summary of tendon and ligament repair

Cell type	Scaffold/Carrier	Model	Result
Tendon cell	Collagen scaffold	Sheep infraspinatus tendon defect model	Damaged tissues have more similar tendon elongation and mechanical strength to natural tendons, providing a better physiological environment for cell proliferation, migration and differentiation [42].
Fibroblast	PGA scaffold	Rat dorsal myofascial model	It is found that fibroblasts are the main cell group involved in tendon regeneration, with increased collagen I content, increased mechanical strength and enhanced tensile properties [187].
Bone marrow mesenchymal stem cells	Acellular tendon matrix scaffold	Rabbit Achilles tendon defect model	Collagen I content increased significantly and had better mechanical strength, stronger tensile properties, stiffness and biocompatibility [188].
Adipose-derived stem cells	Hydrogels containing GDF5 and PDGF	Rabbit tendon defect model	Tendon differentiation was promoted, the growth of fibrous tissue was good, and the mechanical properties were similar to normal tissue [187].
Tendon-derived stem cells	Gelma carrier	Rat tendon defect model	Collagen fibers were arranged better; fibrosis was reduced, mild inflammatory reaction and tendon healing was promoted [180].
Periosteal progenitor cells	Injectable hydrogel made from PEGDA	Rabbit infraspinatus tendon defect model	The number and length of new collagen fibers increased, fibrocartilage adhesion increased, and osteoblast proliferation was good. Bone mineralization and fibrocartilage maturity were also higher [182].

Adipose stem cells (ASCs) induced by growth differentiation factor 5 (GDF-5) promote tissue regeneration and differentiation [181]. Chen *et al*. [182] combined the ASCs cell sheet stimulated by GDF-5 with a nano-yarn scaffold (NNs). The upregulation of Smad2/3 protein and Smad1/5/9 phosphorylation was found to promote the differentiation of tendons, the growth of fibrous tissue was good, and the mechanical properties were similar to those of normal tissues. These findings indicate that the ASCs cell sheet NNs complex is an excellent biological carrier for tendon tissue regeneration. Exosomes are extracellular vesicles stimulating tendon regeneration in stem cells, acting on cells through endocytosis or receptor–ligand interactions [183]. Exosomes are also an essential paracrine factor in stromal cells [184]. Liu *et al*. [185] isolated exosomes from mesenchymal stromal cells (ADSCs). The hydrogel, as a carrier, was combined with an exocrine system. As a result, ADSC-Exos was found to activate the SMAD2/3 and SMAD1/5/9 pathways, promote the proliferation and differentiation of TSCs, and play an important role in inhibiting inflammation. This provides a new approach to the treatment of tendon injuries [186].

## Summary and future perspectives

This article highlights recent advances in tissue regeneration in the application of electrostatic spinning, hydrogel materials, growth factors and cells to tendon regeneration for the repair of tendon tissue. Using drugs, nanoparticles and mineralization gradients enrich scaffolds’ bioactivity and cellular differentiation prepared by electrostatic spinning. Improving scaffolds’ multiscale morphology and mechanical properties is still necessary, and the current fibrous structures are still far from being natural tissues. By integrating additive manufacturing, 3D printing, electrospray and electrospinning, it is possible to improve the balance between electrostatic spinning porosity and mechanical strength. An ideal environment for cell proliferation and differentiation can be achieved by combining mineralization gradients and mechanical properties that approach those of natural tissue. It is necessary to develop an injectable hydrogel with mechanics, tissue damage-specific binding and disease response for future T/L applications. Combining multiple biomaterials will also enable more precise and personalized treatment. Hydrogels can support mechanical repair and biological regeneration through an optimal combination of soft matrix materials and fibrous structures. Moreover, a deeper understanding of the internal regulatory mechanisms of cells, growth factors and scaffolds *in vivo* to control inflammation and neovascularization will significantly impact T/L repair surgery. Many combinations of geometries of customizable T/L materials can be explored, which will result in the development of more candidate materials for ruptured T/L repair and regeneration. They have minimal foreign body reaction and desired tissue growth and can be observed using conventional imaging techniques. Future research should focus on finding the best combination of technologies for treating tendon and ligament injuries, such as cells, growth factors, hydrogels and scaffolds, combined with discoveries in the field of materials, to develop new materials that are better suited for soft tissue.

## Funding

This study was supported by the National Key Research and Development Program of China (2020YFA0908200).

*Conflicts of interest statement*. None declared.
